# Characterizing the Role of Cell-Wall β-1,3-Exoglucanase Xog1p in *Candida albicans* Adhesion by the Human Antimicrobial Peptide LL-37

**DOI:** 10.1371/journal.pone.0021394

**Published:** 2011-06-21

**Authors:** Pei-Wen Tsai, Cheng-Yao Yang, Hao-Teng Chang, Chung-Yu Lan

**Affiliations:** 1 Institute of Molecular and Cellular Biology, National Tsing Hua University, Hsinchu, Taiwan; 2 Division of Animal Medicine, Animal Technology Institute Taiwan, Miaoli, Taiwan; 3 Graduate Institute of Molecular Systems Biomedicine, China Medical University, Taichung, Taiwan; 4 Graduate Institute of Clinical Medical Science, China Medical University, Taichung, Taiwan; 5 Graduate Institute of Basic Medical Science and PhD Program for Aging, China Medical University, Taichung, Taiwan; 6 Department of Life Science, National Tsing Hua University, Hsinchu, Taiwan; Institute of Developmental Biology and Cancer Research, France

## Abstract

*Candida albicans* is the major fungal pathogen of humans. Its adhesion to host-cell surfaces is the first critical step during mucosal infection. Antimicrobial peptides play important roles in the first line of mucosal immunity against *C. albicans* infection. LL-37 is the only member of the human cathelicidin antimicrobial peptide family and is commonly expressed in various tissues, including epithelium. We previously showed that LL-37 significantly reduced *C. albicans* adhesion to plastic, oral epidermoid OECM-1 cells, and urinary bladders of female BALB/c mice. The inhibitory effect of LL-37 on cell adhesion occurred via the binding of LL-37 to cell-wall carbohydrates. Here we showed that formation of LL-37–cell-wall protein complexes potentially inhibits *C. albicans* adhesion to polystyrene. Using phage display and ELISA, we identified 10 peptide sequences that could bind LL-37. A BLAST search revealed that four sequences in the major *C. albicans* cell-wall β-1,3-exoglucanase, Xog1p, were highly similar to the consensus sequence derived from the 10 biopanned peptides. One Xog1p-derived peptide, Xog1p_90–115_, and recombinant Xog1p associated with LL-37, thereby reversing the inhibitory effect of LL-37 on *C. albicans* adhesion. LL-37 reduced Xog1p activity and thus interrupted cell-wall remodeling. Moreover, deletion of *XOG1* or another β-1,3-exoglucanase-encoding gene *EXG2* showed that only when *XOG1* was deleted did cellular exoglucanase activity, cell adhesion and LL-37 binding decrease. Antibodies against Xog1p also decreased cell adhesion. These data reveal that Xog1p, originally identified from LL-37 binding, has a role in *C. albicans* adhesion to polystyrene and, by inference, attach to host cells via direct or indirect manners. Compounds that target Xog1p might find use as drugs that prevent *C. albicans* infection. Additionally, LL-37 could potentially be used to screen for other cell-wall components involved in fungal cell adhesion.

## Introduction

*Candida albicans* is an opportunistic pathogenic yeast that commonly colonizes mucosal surfaces and can cause severe blood infections in immunocompromised individuals [1], [2]. Interaction between *C. albicans* and epithelial cells is necessary for disease development and progression. Initially, *C. albicans* adheres to and colonizes epithelial cell surfaces prior to invading and disrupting the cells [3]. *C. albicans* expresses various cell-wall components that facilitate cell adhesion [4]. As a counter to *C. albicans* infection, epithelial cells first produce antimicrobial compounds, e.g., defensins, cathelicidins, and histatins, which can kill the fungus or prevent its adhesion to host cells [5], [6], [7].

Cathelicidins are antimicrobial peptides that contain a highly conserved cathelin domain and a highly variable cathelicidin domain [8]. For human cathelicidin, proteinase-3 cleaves its C-terminal region, thereby generating the mature, active 37-residue antimicrobial peptide LL-37 [9] that contains two leucine residues (LL) at the N terminus [10]. LL-37 is positively charged at neutral pH, contains many hydrophobic and basic residues, and is α-helical. These properties allow LL-37 to bind and disrupt the negatively charged membranes of pathogens, leading to cell death [11], [12]. LL-37 is produced by neutrophils, macrophages, mucosal epithelial cells, and keratinocytes [13], which suggests that it is part of the innate immunity system, protects against infection, and participates in the inflammatory response [14]. In addition to its antimicrobial and cytotoxic activities, LL-37 also functions in leukocyte chemotaxis, endotoxin neutralization, inhibition of microbial adhesion, and wound healing at epithelial surface [15], [16], [17], [18]. LL-37 acts by interacting with microbial cell walls, the plasma membrane, cellular proteins, and DNA [7], [19], [20], [21].

The *C. albicans* cell wall is a dynamic and highly regulated structure that forms the outermost layer of the cell, thus maintaining cell shape and integrity and interacting with host cells and the surrounding environment [22]. It contains the polysaccharides glucan, chitin, and mannans, which form the outer fibrillar layer. The mannans are often conjugated to proteins or lipids and represent 35–40% of the total cell-wall polysaccharides [23], [24]. Cell-wall proteins (CWPs) function during cell-wall assembly and remodeling, adhesion to a host or an abiotic surface, biofilm formation, invasion of epithelia, and as part of the escape mechanism from the host immune system [25], [26], [27], [28]. Except for certain heat-shock proteins and glycolytic enzymes, most external coat of CWPs are glycosylphosphatidylinositol (GPI) proteins that are often highly mannosylated and phosphorylated [25], [29], [30]. In *Saccharomyces cerevisiae*, disulfide bridges of the external protein coat affect cell wall permeability [31]; this may be also the case in *C. albicans*, suggesting that GPI-CWPs might be interconnected by disulfide bonds [25]. In addition, CWPs can be released from intact cells by reducing agents [32], it is assumed that CWPs are linked to other CWPs by disulfide bridges [24]. The cell-wall enzymes include glucanases, chitinases, peptidases, and glycotransferases that are involved in cell-wall synthesis and remodeling, thus providing flexibility and strength to the cell wall during cell growth or lysis in response to a stress [33], [34], [35]. Because the structural complexity of the cell-wall components is crucial to *C. albicans* physiology, targeting the integrity or functions of its cell wall is an excellent way to interfere with infection processes, such as cell adhesion [36].

β-1,3-glucan and β-1,6-glucan are the major structural components of the *C. albicans* cell wall and account for 40% and 20% of the cell-wall components, respectively. The flexible, three-dimensional cell-wall network is composed of β-1,3-glucan [24]. In *S. cerevisiae*, glucanases remodel the glucan network by nicking the glucans and introducing branch points [37]. In *C. albicans*, three related exo-β-1,3-glucanases, Xog1p, Exg2p, and Spr1p, hydrolytically remove glucose from the ends of cell-wall glucans [35], [38], [39]. The purpose of *C. albicans* Spr1p has yet to be determined, although in *S. cerevisiae* it is specifically expressed during sporulation [40], [41]. Exg2p is present during *C. albicans* cell-wall regeneration [40], [41]. Xog1p is the major exo-1,3-β-glucanase associated with the *C. albicans* periplasmic cell wall. It is a non-glycosylated protein with a molecular mass of ∼45 kDa, which is also the approximate mass of Exg2p and Spr1p, and its sequence is 58% identical to that of *S. cerevisiae* Exg1p [42], [43], [44]. Although Xog1p is responsible for cell-wall construction and remodeling [45], it may have additional roles that need to be delineated.

For the study reported herein, we identified and characterized an interaction between LL-37 and *C. albicans* Xog1p that adversely affects *C. albicans* adhesion. We found that LL-37 binding to Xog1p diminished the glucanase activity of Xog1p and significantly reduced *C. albicans* attachment to polystyrene. Moreover, Xog1p itself was shown to be directly or indirectly involved in *C. albicans* adhesion. Therefore, drugs that target Xog1p might be used to prevent *C. albicans* adhesion. Furthermore, LL-37 could perhaps be used to screen for other cell-wall molecules involved in *C. albicans* adhesion to substrata.

## Results

### Binding of LL-37 to *C. albicans* CWPs

**Figure 1:**
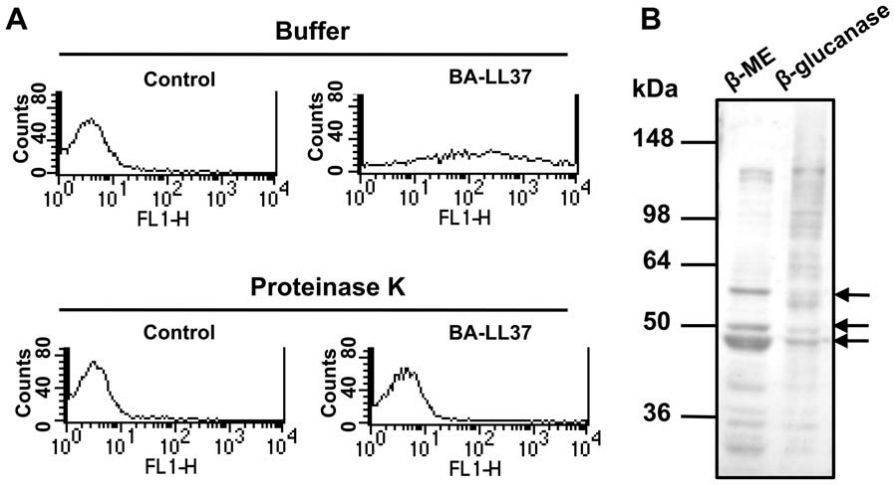
Binding of LL-37 to *C. albicans* cell-wall proteins. (A) Flow cytometry showing that BA-LL37 bound to *C. albicans*. Cells were treated with 1 mg/ml proteinase K (lower panels) or were not treated (upper panels) prior to incubation with 10 µg BA-LL37. The fluorescence intensity (FL1-H) of SA-DTAF that was associated with the cells via binding to BA-LL37 was measured to determine the amount of BA-LL37 bound to cells. The results are representative of two independent experiments that gave similar results. (B) Extracts prepared by fractionation of *C. albicans* cell-wall proteins using β-ME and β-glucanase. The proteins in the extracts were separated by SDS-PAGE and transferred to a polyvinylidene difluoride membrane. The membranes were probed with BA-LL37 and visualized with HRP–conjugated streptavidin. Arrows indicate the three major cell wall proteins bound by LL-37. The positions and values of molecular mass standards are indicated. Data are representative of three independent experiments that gave similar results.

In our previous study we found that LL-37 inhibited the adhesion of *C. albicans* to polystyrene by binding to cell-wall polysaccharides, in particular, mannan [21]. However, after removal of ∼50% of the carbohydrates from the cell wall, the amount of LL-37 bound to *C. albicans* was reduced by only ∼30% as compared with the amount bound to control (non-deglycosylated) cells [21]. In addition to the high carbohydrate content of the *C. albicans* cell wall, proteins represent 20∼30% of the total mass [33]. Therefore, we could not exclude the possibility that LL-37 interacts with CWPs. To test this hypothesis, the binding of biotinylated LL-37 (BA-LL37) to *C. albicans* was assessed. For the removal of proteinacous layer, *C. albicans* cells were preincubated with proteinase K followed by treatment with BA-LL37. BA-LL37 bound to these cells was assessed using flow cytometry in conjunction with SA-4,6-dichlorotriazinyl aminofluorescein (SA-DTAF) detection [21]. Given the fluorescence intensities measured (Fig. 1A, upper panels), it was apparent that BA-LL37 bound to *C. albicans*. In contrast, after removing cell-wall proteins with proteinase K, the binding of BA-LL37 was almost abolished (Fig. 1A, lower panels). To substantiate these results, the CWPs were isolated, and LL-37 binding was demonstrated by far-western blotting using BA-LL37 as the probe. Several CWPs from an extract prepared from β-mercaptoethanol (β-ME)-treated cells bound BA-LL37, particularly two proteins with molecular masses of 45–50 kDa and a third protein with a mass of ∼60 kDa (Fig. 1B). However, LL-37 did not appear to specifically bind proteins in an extract prepared from β-glucanase-treated cells (Fig. 1B). Therefore, only the β-ME extract was examined further. These results indicate that LL-37 interacts with both cell-wall polysaccharides [21] and proteins.

### Xog1p: an LL-37-binding target identified by phage-display biopanning

**Figure 2:**
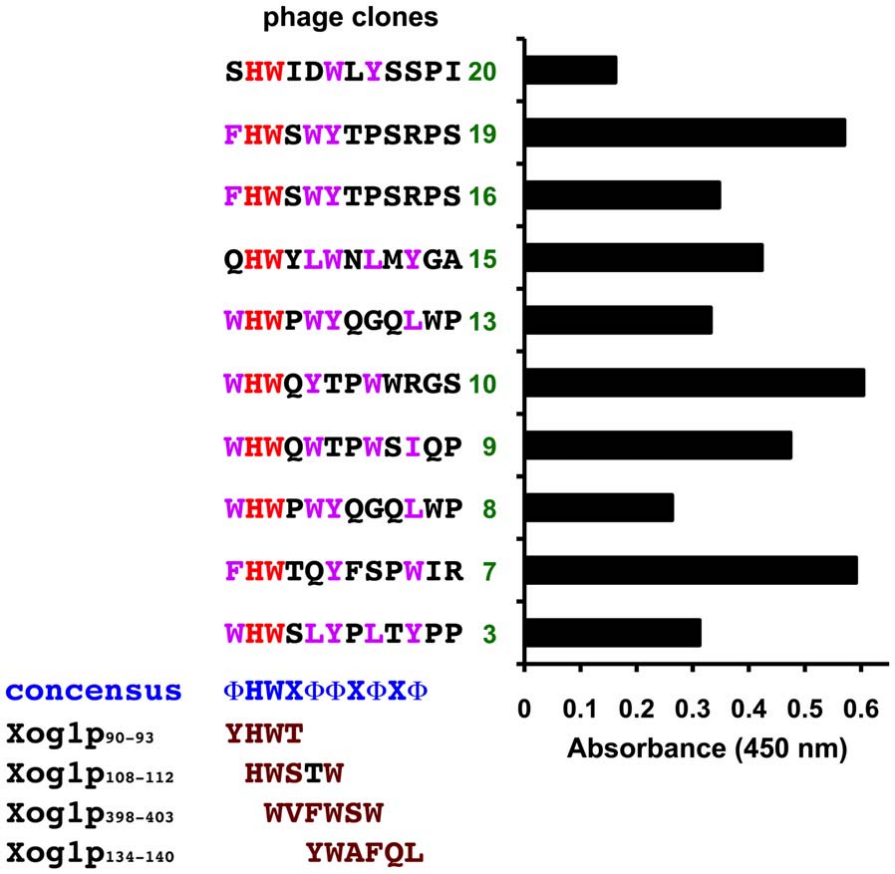
Identification of potential LL-37-binding peptides by phage display. Twenty phage clones were isolated after three rounds of biopanning. The interaction between 10 of the phage-displayed peptides and LL-37 was confirmed by ELISA. The phage samples were added into LL-37-pre-coated plates for binding, and after removing unbound phages, the phage/LL-37 complexes were stained with anti-M13 and detected using an ELISA reader. Those sequences for which the A_450_ was >0.05 are shown in the figure. The 10 sequences are aligned. Identical residues are in red, residues with similar physicochemical properties are in magenta, and the consensus sequence is in blue. X indicates any amino acid, and Φ indicates a hydrophobic residue. The consensus sequence is aligned with four sequences found in Xog1p at the bottom of the figure. Residues in the peptides that are homologous with or identical to residues in the consensus sequence are in brown.

To identify which *C. albicans* CWPs were targeted by LL-37, phage-display biopanning was performed using a linear dodecapeptide library and LL-37. After three rounds of biopanning, 20 phage clones had been amplified and were characterized by DNA sequencing and ELISA, the results of which indicated that 10 clones could bind LL-37. Although the extent of LL-37 binding varied, these clones contained the consensus peptide sequence ΦHWXΦΦXΦXΦ, where Φ is a hydrophobic residue and X represents any residue (Fig. 2). This consensus sequence was used in candidates to identify potential LL-37-binding proteins in the *Candida* Genome Database (www.candidagenome.org). Among the potential binding targets identified, Xog1p was chosen for further characterization because it has a molecular mass of ∼45 kDa, which is similar to that for one of the CWPs identified by far-western blotting (Fig. 1B) and because Xog1p is the major exo-β-1,3-glucanase involved in *C. albicans* cell-wall remodeling and assembly [45]. Interestingly, four peptide sequences in Xog1p matched well with those of the phage-display and were therefore predicted to be possible LL-37 recognition and binding sites (Fig. 2). These data suggested that Xog1p is a potential LL-37 target.

### LL-37 associates with Xog1p

**Figure 3:**
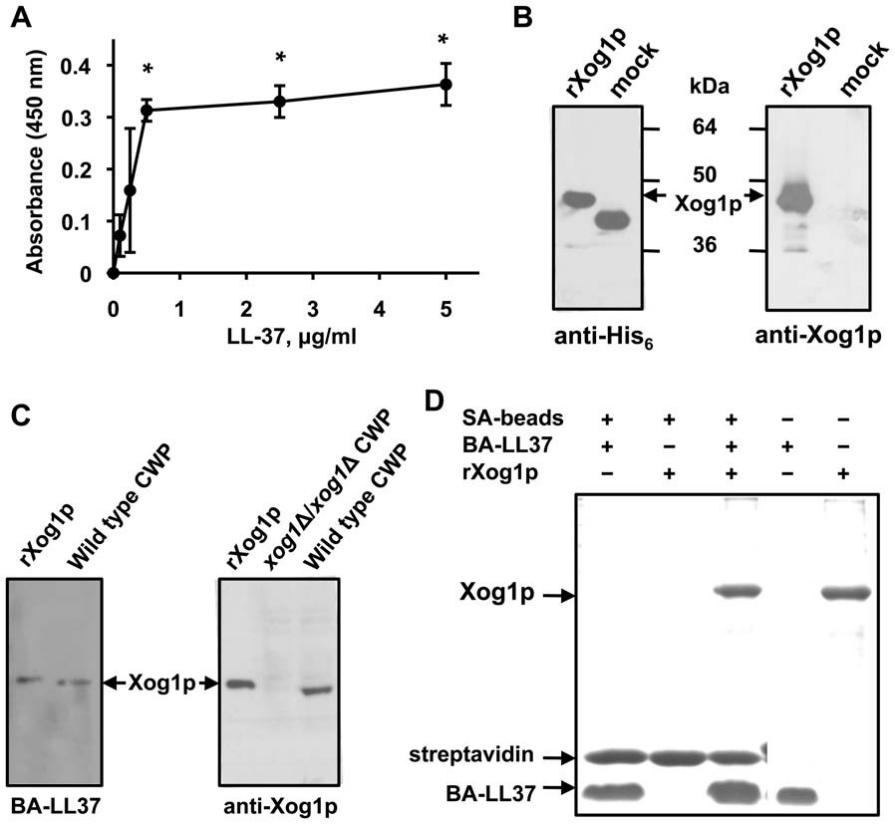
Expression of full-length Xog1p and its interaction with LL-37. (A) ELISA for the binding of Xog1_90–115_ to LL-37. Wells were coated with 5 µg of Xog1_90–115_ and then incubated with different concentrations of BA-LL37 at room temperature for 1 h. Binding was detected with SA-HRP. All assays were performed in triplicate and carried out four times. A representative experiment is shown. Each value is the mean ± the SD of the absorbance recorded for one experiment. The statistical significance for the binding of treated vs. control wells was determined using Student's *t*-test (*, *p*<0.05). (B) Analysis of rXog1p and anti-Xog1p serum. Western blots of purified rXog1p using anti-Xog1p (right panel) or anti-His_6_ (left panel) as probe. Mock is His-tagged recombinant d-amino acid oxidase. The positions of molecular mass standards (kDa) are indicated. (C) Western blots of rXog1p and the β-ME cell-wall extract (CWP) from wild type and the *xog1* mutant strain. rXog1p and CWP were probed with BA-LL37 (left panel) or anti-Xog1p (right panel). (D) Pull-down assay for LL-37-rXog1p binding. Purified rXog1p was incubated with BA-LL37 and streptavidin-agarose beads for 2 h at room temperature. The complexes were isolated by centrifugation, washed to remove non-specific BA-LL37-binding proteins, and subjected to SDS-PAGE. rXog1p was visualized by Coomassie Blue staining.

Because there were four potential LL-37 recognition/binding sequences in Xog1p (Fig. 2), we also mapped these sequences to the Xog1p three-dimensional structure [46]. Two of the peptide sequences (residues 90–93 and 108–112, Fig. 2) are at the surface [46]. A short peptide containing residues 90–115 (Xog1_90–115_) was synthesized and used in the LL-37-binding/ELISA assay. LL-37 bound Xog1_90–115_ in a dose-dependent and saturable manner (Fig. 3A).

To characterize LL-37-Xog1p binding, we expressed a soluble His-tagged recombinant Xog1p (rXog1p) and produced rat polyclonal antibodies against Xog1p for use in western blotting. The expression and purification of rXog1p was confirmed by western blotting with anti-His_6_ (Fig. 3B, left panel). Moreover, rXog1p had exoglucanase activity (data not shown), and anti-Xog1p recognized rXog1p (Fig. 3B, right panel). β-ME cell-wall extracts were also examined by western blotting. BA-LL37 bound to a protein of 45 kDa that co-migrated with rXog1p and was recognized by anti-Xog1p (Fig. 3C). BA-LL37 was then used as bait in pull-down assays under native conditions. BA-LL37 pulled down full-length rXog1p, and this complex was distinguishable from BA-LL37 (Fig. 3D). Together, these results strongly suggested that Xog1p is an LL-37-binding target that resides on the *C. albicans* cell wall.

### Roles of Xog1p in LL-37-mediated inhibition of *C. albicans* adhesion

**Figure 4:**
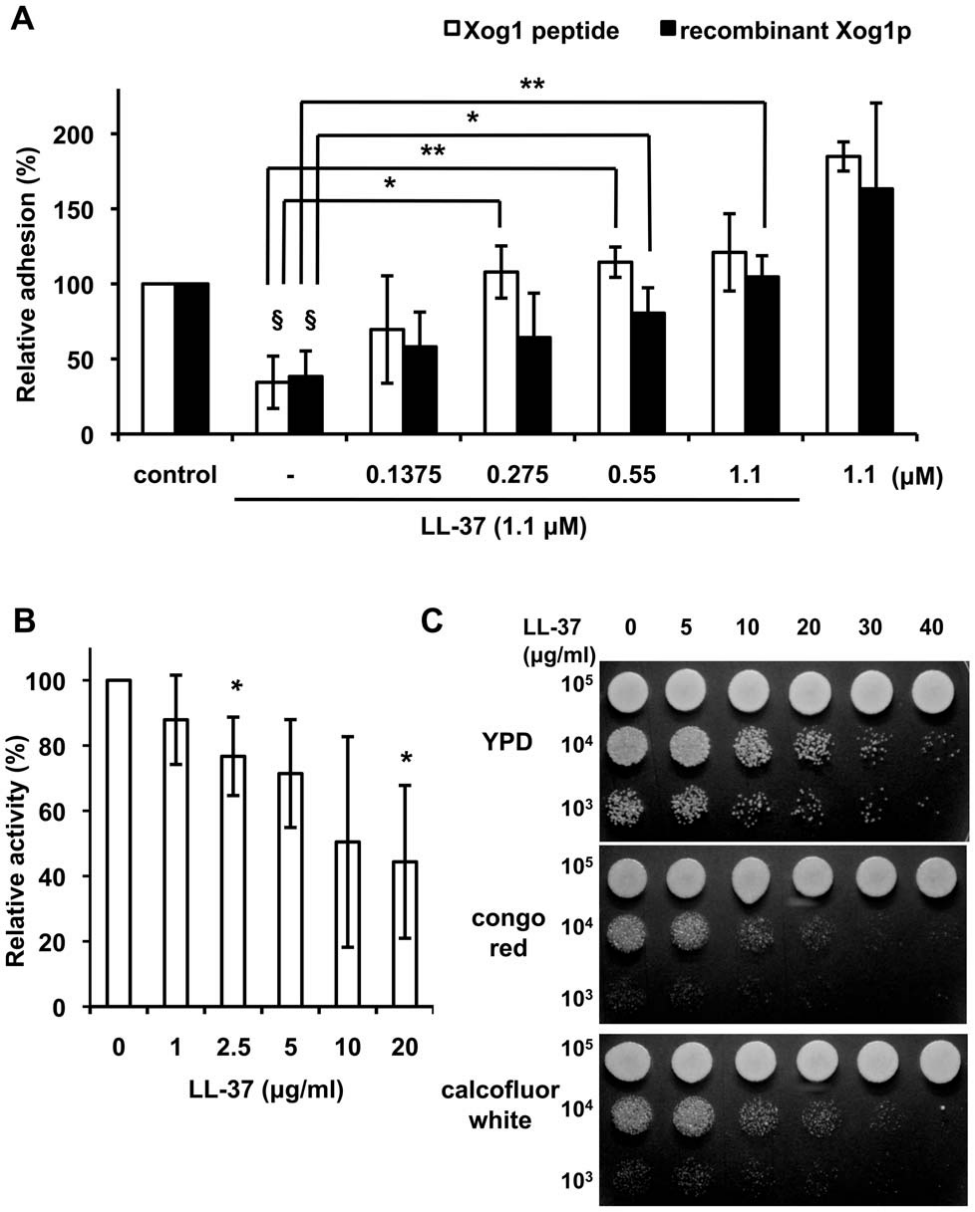
Xog1p plays a role in LL-37-mediated inhibition of *C. albicans* adhesion. (A) Assessment of the abilities of Xog1_90–115_ (white bars) and rXog1p (black bars) to rescue LL-37-mediated inhibition of *C. albicans* adhesion to polystyrene. Cells were incubated with 1.1 µM LL-37 and different concentrations of Xog1_90–115_ or rXog1p. Reduction of XTT was measured to assess the number of cells that adhered to the polystyrene wells. The data were normalized to the corresponding control experiment (no LL-37) and are reported as a percentage. The right-most bars report the results for cells incubated with only Xog1_90–115_ or rXog1p. Each result is the average of three experiments, each performed in triplicate. The two-tailed Student's *t*-test was used to determine the statistical significance of the data (§, *p*<0.01 for LL-37-treated cells vs. control cells; *, *p*<0.05 and **, *p*<0.01 for LL-37-treated cells in the presence of Xog1_90–115_ or rXog1p vs. cells treated only with LL-37). (B) Exoglucanase activity in cell-free extracts of wild type *C. albicans*. Cells were incubated with different concentrations of LL-37 in RPMI-1640 at 37°C for 30 min, and then cell-free extracts were prepared. Exoglucanase activity was assayed using the model substrate PNPG and expressed as a percentage. Assays were performed in quadruplicate. (C) Susceptibilities of LL-37-treated cells to cell-wall-interrupting agents were demonstrated by spot assays on YPD plates. Upper panel, controls; middle panel, 15 µg/ml congo red; bottom panel, 10 µg/ml calcofluor white. *C. albicans* was treated with different concentrations of LL-37, and ten-fold serial dilutions of the cells were spotted onto the plates. These results are representative of three independent experiments that gave similar results.

We previously showed that LL-37 inhibits *C. albicans* adhesion to polystyrene by binding to the cells with increasing LL-37 concentrations starting from 0.1 to 20 µg/ml [21]. We tested if Xog1_90–115_ could also block LL-37 binding to Xog1p and thereby compensate reduction of cell adhesion. LL-37 was mixed with Xog1_90–115_, added to *C. albicans* cells, and then the 2,3-bis-(2-methoxy-4-nitro-5-sulfophenyl)-2H-tetrazolium-5-carboxanilide (XTT)-reduction assay was performed. At 5 µg/ml (∼1.1 µM) LL-37, the adhesion inhibition mediated by LL-37 was ∼70% (Fig. 4A) which was consistent with our previous finding [21]. Xog1_90–115_ prevented LL-37 inhibition of cell adhesion in a dose-dependent manner when the concentration of Xog1_90–115_ was at least 0.1375 µM (Fig. 4A). Therefore, LL-37 may interact with cell-wall Xog1p and thereby reduce *C. albicans* adhesion. Additionally, rXog1p was tested in the *C. albicans* adhesion assay to determine if it could reverse the inhibitory effect of LL-37. As expected, LL-37 reduced the adhesion of *C. albicans* to polystyrene, whereas rXog1p rescued the LL-37-induced inhibition of cell adhesion (Fig. 4A).

Xog1p is the major *C. albicans* cell-wall exoglucanase [45]. Disruption of *C. albicans XOG1*, which encodes Xog1p, alters the cell-wall composition and changes the susceptibility of *C. albicans* to antifungal agents that inhibit β-(1,3)-glucan synthesis [45] or chitin biosynthesis [47]. We therefore considered the possibility that LL-37-mediated inhibition of cell adhesion may be the result of disrupted cell-wall remodeling. Cell adhesion to a substratum may alter the framework of the cell wall. Therefore, it was important to determine if Xog1p activity could be affected by LL-37. We assayed the *in vitro* exoglucanase activity in cell-free extracts from logarithmically growing *C. albicans*. Xog1p activity decreased as the LL-37 concentration increased (Fig. 4B), implying that the assembly of the cell-surface glucan network might be altered by a decrease in Xog1p activity, which might thereby affect cell adhesion. LL-37-induced inhibition of cell-wall remodeling was reflected in increased susceptibilities to different agents that interrupt cell-wall integrity. The viability of LL-37-treated cells on agar plates that contained such an agent, i.e., congo red and calcofluor white, decreased in comparison with those cultured in the absence of such agents (Fig. 4C), indicating that cell-wall remodeling had been altered and possibly inhibited by LL-37. Therefore, the binding of LL-37 to Xog1p might indirectly interfere with cell adhesion by interrupting cell-wall assembly.

### Xog1p is involved in *C. albicans* adhesion

**Figure 5:**
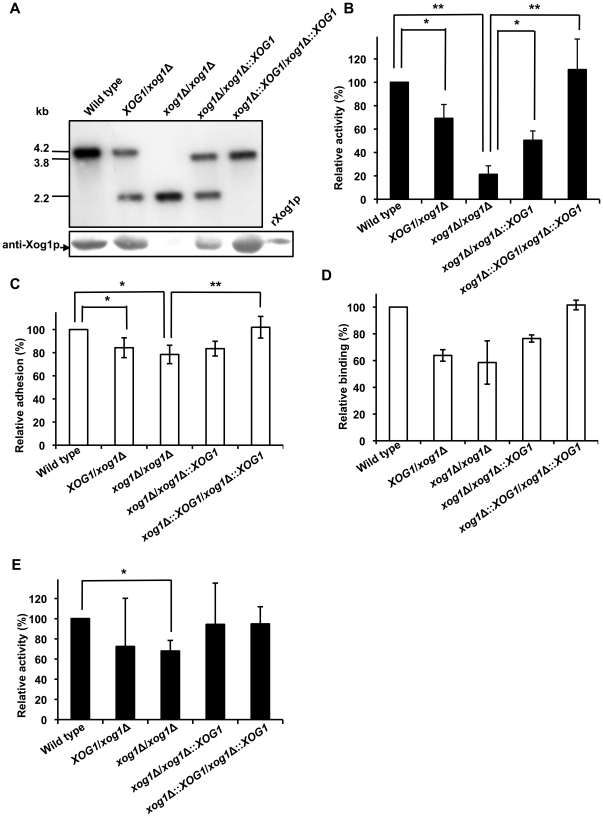
*XOG1* deletion reduces *C. albicans* adhesion to polystyrene and decreases cell association with LL-37. (A) Analysis of the construction of the *XOG1* deletion and reintegrated strains. For Southern blotting (upper panel), enzyme-digested chromosomal DNA was subjected to agarose gel electrophoresis and transferred to a nylon membrane. The membrane was probed with a 0.44-kb, P^32^-labeled DNA fragment containing a *C. albicans XOG1* upstream sequence. For western blotting (lower panel), a β-ME cell-wall extract and rXog1p were used. Equal volumes of protein were subjected to SDS-PAGE and then detected with rat polyclonal anti-Xog1p. (B) Exoglucanase activity assay for various *C. albicans* strains. Overnight cultures were collected and assayed using PNPG as the substrate. All assays were carried out three times. The two-tailed Student's *t*-test was used to determine the statistical significance of the data; *, *p*<0.05; **, *p*<0.01. (C) Adhesion of *C. albicans XOG1*-mutant strains. Each strain was suspended in RPMI-1640 medium and incubated at 37°C for 30 min in polystyrene wells. After washing, cells that remained in the wells were assayed by XTT reduction. Each result is the mean ± SD of four independent assays. The two-tailed Student's *t*-test was used to determine the statistical significance of the data; *, *p*<0.05; **, *p*<0.01. (D) Comparison of LL-37 binding to wild type, *XOG1*-deletion, and *XOG1*-reintegrated strains. Each strain was individually mixed with BA-LL37 in PBS at 4°C overnight, and the binding of LL-37 to the cells was measured by flow cytometry using streptavidin-conjugated 4,6-dichlorotriazinyl aminofluorescein. Each result is the mean ± SD of two independent assays. (E) Exoglucanase activity in cell extracts from various *C. albicans* strains. Logarithmically growing cultures were collected and assayed using PNPG. All assays were carried out three times. The two-tailed Student's *t*-test was used to determine the statistical significance of the data; *, *p*<0.05.

Given that LL-37 inhibited cell adhesion by binding Xog1p, Xog1p itself might be involved in the adhesion process via direct or indirect manners. To further explore the role of Xog1p in cell adhesion, *XOG1* heterozygous- and homozygous-deletion mutants and *XOG1*-reintegrated strains were generated by the *SAT1*-flipper method [48]. Polymerase chain reaction (PCR; data not shown) and Southern and western blotting (Fig. 5A) showed that the strain constructs were as expected. For Southern blotting, genomic DNA was digested with *Pst*I and *EcoR*I and hybridized to a probe that contained the 0.4-kb P^32^-labeled DNA fragment upstream of *XOG1*. Nucleotide fragments of 4.2 and 3.8 kb, which were the expected lengths for the wild type and reintegrated *XOG1* fragments, and a 2.2-kb band, which was the expected length for the deleted *XOG1* fragment, were found in the Southern blot (Fig. 5A, upper panel). For western blotting, CWPs were isolated from *C. albicans* grown in RPMI-1640 medium at 37°C for 30 min. Xog1p from wild type cells was detected by anti-Xog1p but was not detected in the extract of the *xog1*Δ/*xog1*Δ strain (Fig. 5A, lower panel and Fig. 3C). Furthermore, we measured exoglucanase activity in the wild type and *XOG1*-deletion strains from overnight cultures using the model substrate *p*-nitrophenyl β-d-glucopyranoside (PNPG) [45]. Deletion of *XOG1* resulted in a significant reduction (∼80%) in the hydrolysis of PNPG compared with wild type cells (Fig. 5B). Interestingly, the *XOG1*/*xog1*Δ and *xog1*Δ/*xog1*Δ::*XOG1* strains displayed an intermediate phenotype (Fig. 5B), indicating that deletion of a single allele decreased glucanase activity.

Because cell adhesion is the first critical step during *C. albicans* infection, we examined the involvement of Xog1p in adhesion. As indicated, homozygous deletion of *XOG1* reduced *C. albicans* adhesion to polystyrene by ∼20% (Fig. 5C). Conversely, the *xog1*Δ/*xog1*Δ::*XOG1* strain adhered to polystyrene to a similar extent as the *XOG1*/*xog1*Δ heterozygous mutant. The strain in which both *XOG1* alleles were reintegrated behaved as the wild type strain did (Fig. 5C). Binding of BA-LL37 to the wild type and the deletion strains was also measured. The heterozygous and homozygous strains showed a 30∼40% decrease in their ability to bind BA-LL37 as compared with the wild type strain. The *XOG1*-reintegrated strain bound BA-LL37 as well as the wild type strain (Fig. 5D). Collectively, these data indicated that Xog1p may play a role in *C. albicans* adhesion and is one of the binding targets of LL-37.

Because deletion of *XOG1* did not completely abolish cell adhesion to polystyrene and cell binding to LL-37, it is possible that compensatory mechanisms were triggered to rescue the *XOG1* deficiency, i.e., when *XOG1* was deleted, other exoglucanases might have been overexpressed. Therefore, exoglucanase activity was measured in cell-free extracts from logarithmic–growth phase wild type, *XOG1*-deletion, and reintegrated strains. Deletion of both *XOG1* alleles resulted in an ∼30% decrease in glucanase activity compared with that of wild type cells (Fig. 5E). During the logarithmic-growth phase, homozygous deletion of *XOG1* did not cause as dramatic a reduction in exoglucanase activity as when the cells were in the stationary phase (∼80% reduction, Fig. 5B), suggesting that other exoglucanases function in the rapid growing cells requiring cell-wall assembly.

**Figure 6:**
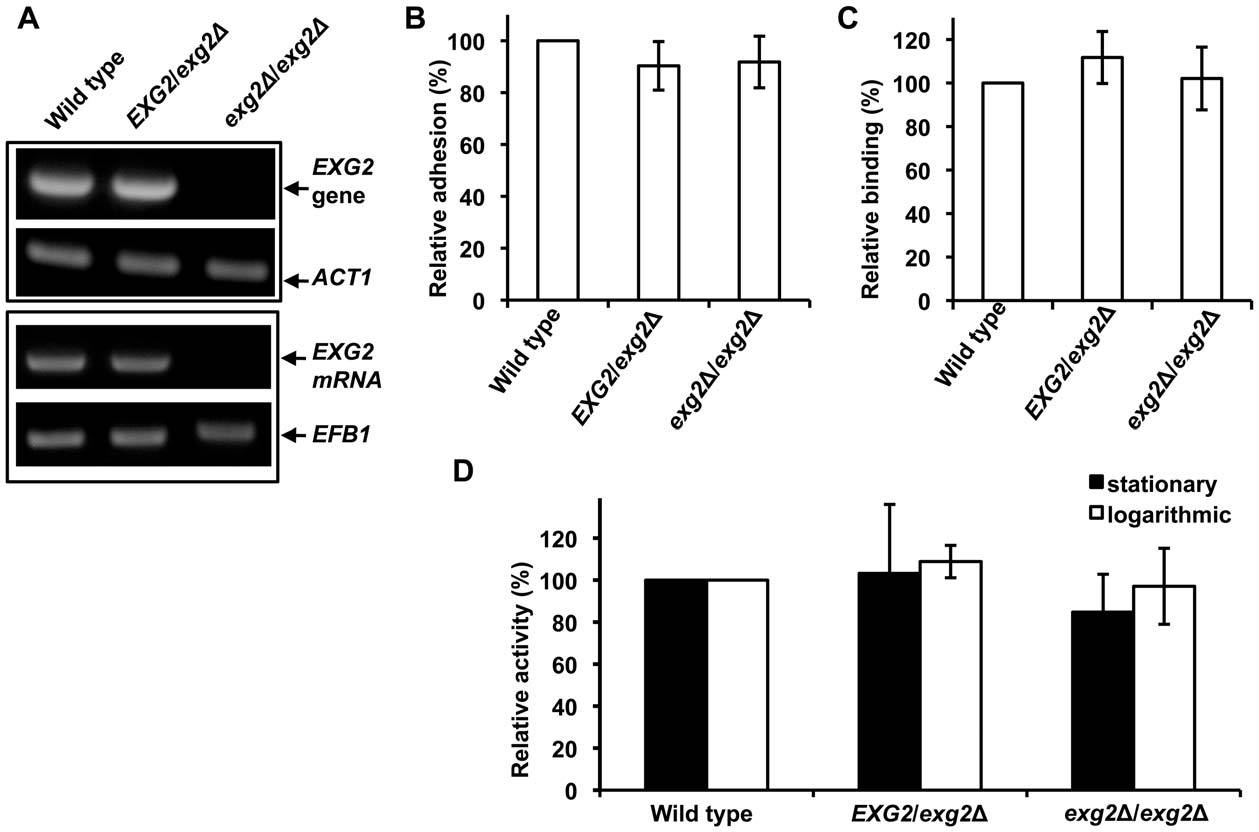
*EXG2* has no significant effect on *C. albicans* cell adhesion or cellular interaction with LL-37. (A) Construction of *EXG2*-deletion strains was verified by PCR (upper panel) and RT-PCR (lower panel). The amplified products of *ACT1* and *EFB1* were used as positive controls. (B) The levels of *C. albicans* adhesion to polystyrene were assayed by measuring reduced XTT. The relative adhesion of a mutant is expressed as a percentage of that found for the wild type strain. Each result is the mean ± SD of four independent assays. (C) Comparison of LL-37 binding to wild type and *EXG2*-deletion strains. Each strain was mixed with BA-LL37 in PBS at 4°C overnight, and binding of LL-37 to the strains was measured by flow cytometry. Each result is the mean ± SD of two independent assays. (D) Exoglucanase activity assays for the wild type and *EXG2*-deletion strains. Cells from logarithmic- or stationary-growth phase were collected, and glucanase activity in the cells and in cell extracts was assayed using the model substrate PNPG. Each result is the mean ± SD of three independent assays.

**Figure 7:**
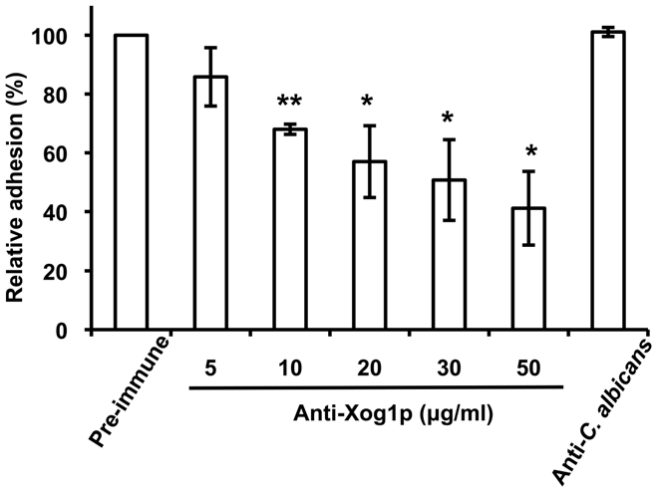
Xog1p plays a role in the adhesion of *C. albicans* to polystyrene. Inhibition of *C. albicans* adhesion to polystyrene by anti-Xog1p. Wild-type *C. albicans* was incubated with the IgG-rich fractions that had been purified from anti-Xog1p, pre-immune and anti-*C. albicans* serum. Each result is the mean ± SD of assays performed in triplicate. The two-tailed Student's *t*-test was used to determine the statistical significance of the adhesion of anti-Xog1p treated vs. mock cells was determined using Student's *t*-test; *, *p*<0.05; **, *p*<0.01.

A search of the *C. albicans* genome database identified an exoglucanase-encoding gene, *EXG2*, which is induced during cell-wall regeneration [35], [41]. To determine if Exg2p, encoded by *EXG2*, is also involved in *C. albicans* cell adhesion, *EXG2*-deletion mutants were constructed and verified by PCR (Fig. 6A, upper panel) and reverse-transcription PCR (Fig. 6A, lower panel). Interestingly, in contrast to the deletion of *XOG1*, *EXG2* deletion did not significantly reduce *C. albicans* adhesion (Fig. 6B) or LL-37 cell binding (Fig. 6C). Deletion of *EXG2* did not reduce glucanase activity (Fig. 6D), in agreement with the cell-adhesion and LL-37-binding data (Fig. 6B and 6C), i.e., Exg2p is not heavily involved in exoglucanase function in adhesion process of *C. albicans*. To exclude a possible compensatory effect in *XOG1* mutant strains after cultivation, a purified IgG-enriched fraction from anti-Xog1p serum was tested for its ability to inhibit the adhesion of wild type cells to polystyrene. We suspect that anti-Xog1p serum might act similarly as LL-37, which inhibits adhesion of *C. albicans* through directly binding with Xog1p or indirectly declining the glucanase activity of Xog1p. The anti-Xog1p serum inhibited ∼60% of the cell adhesion to polystyrene compared with pre-immune or anti-*C. albicans* serum (Fig. 7). Together, these results suggested that Xog1p is targeted by LL-37 and is involved in *C. albicans* adhesion.

## Discussion

Antimicrobial peptides are vital for epithelial host defense. As the only antimicrobial peptide of the human cathelicidin family, LL-37 defends the epithelium against microbes by several different ways, including its constitutive expression by epithelial cells in the absence of microbes, increasing its production and secretion in the presence of microbes, directly killing nearby microbes, inhibiting microbial adhesion to the epithelium, and recruitment of neutrophils to secrete more LL-37 complementary to the epithelial sources [49]. To date, the diverse effects of LL-37 have focused on bacterial infection. Foschiatti *et al.* [50] demonstrated an interaction between LL-37 and bacterial exopolysaccharides. Bergsson *et al.* reported that LL-37 associates with glycosaminoglycans in lung fluid from cystic fibrosis patients [51]. These carbohydrates/LL-37 complexes neutralize the antimicrobial activity of LL-37 [50], [51]. Although it is well documented that epithelium-derived LL-37 substantially protects against bacterial infection, less is known about the action of LL-37 against fungal pathogens.

We recently reported the first study showing that LL-37 interferes with fungal adhesion by documenting that sublethal doses of LL-37 inhibit *C. albicans* adhesion to polystyrene, oral epithelial cells, and mouse urinary bladder [21]. We provided evidence that LL-37 partially inhibits *C. albicans* adhesion by interacting with cell-wall carbohydrates and suggested that LL-37 may also interact with proteins, which are a major component of the *C. albicans* cell wall. To test this hypothesis, for the study reported herein, we used phage display and other approaches to identify *C. albicans* surface proteins that can bind LL-37. We demonstrated that LL-37 binds Xog1p (Fig. 3). The binding of antimicrobial peptides to microbial surface proteins has been reported previously. In the anaerobic bacteria *Finegoldia magna*, the *F. magna* adhesion factor binds LL-37 and blocks its killing activity, allowing *F. magna* to proliferate in humans [52]. LL-37 also interacts with *Escherichia coli* curli fibers, which inhibits their self-polymerization and thereby prevents *E. coli* cell adhesion to substrata and biofilm formation [53]. Human β-defensin-3 binds to immobilized recombinant hemagglutinin B, a nonfimbrial adhesin from *Porphyromonas gingivalis*, thereby preventing adhesion of the bacterium to host tissues [6], [54]. Moreover, the *C. albicans* cell-wall Ssa1p and Ssa2p chaperones bind and help import of salivary histatin 5, which is required for toxicity [55].

*C. albicans* expresses surface glycans and proteins that act as adhesins and other binding proteins to contract with substrates [4]. Several *C. albicans* adhesins are reported, including the Als and Hwp1 proteins [56]. Notably, many cell-wall-associated enzymes are also involved in cell adhesion. Camp65, a 65-kDa mannoprotein, is believed to be a β-endoglucanase, is a possible target of the host immune response, and has adhesive properties [57]. The endoglucanase activity of Camp65 contributes to cell-wall degradation and remodeling [58]. The secreted aspartate proteinases (Saps) of *C. albicans*, Sap1p, Sap2p, and Sap3p, are involved in the adhesion to buccal epithelial cells [59]. Xog1p is secreted into the cell wall where it acts to breakdown β-1,3-glucan during cell-wall remodeling [44]. In our study, interaction of LL-37 and cell-wall Xog1p reduced *C. albicans* adhesion to polystyrene (Fig. 4A), possibly because LL-37 indirectly decreased Xog1p activity (Fig. 4B), thereby preventing cell-wall assembly (Fig. 4C). Moreover, the *xog1*Δ/*xog1*Δ strain had reduced adhesion to polystyrene (Fig. 5C) and attenuated exoglucanase activity (Fig. 5E). Anti-Xog1p serum inhibited ∼60% of the *C. albicans* adhesion to polystyrene (Fig. 7). Together, these results strongly suggest that Xog1p may be directly or indirectly involved in the process of *C. albicans* adhesion. Torosantucci *et al.* suggested that an antibody against β-1,3-glucan might be used to inhibit fungal growth and adhesion [60]. In our previous study, we showed that LL-37 inhibited cell adhesion by binding to cell-wall carbohydrates, e.g., glucan [21]. On the basis of our studies and those of others, it appears that β-1,3-glucan is involved in *C. albicans* adhesion to host cells. Our new results suggest that the cell-wall glucan network may be interfered by LL-37 via the inhibition of Xog1p exoglucanase activity (Fig. 4B and 4C). We thus hypothesized that dysfunction of exoglucanase can not only alter the glucan composition of the cell wall (unpublished data), but also affect glycosylation of other cell wall proteins required for adhesion, leading to impair cell adhesion.

Although the activity of Xog1p decreased in the presence of LL-37, the mechanism responsible for the decrease has not been delineated. Notably, two structural Xog1p loops have been proposed as the sites that bind cell-wall glucan during remodeling [61]. Interestingly, three of the four Xog1p sequences identified by searches in CGD after biopanning (Fig. 2) are located near to these loops. The two catalytically important glutamate residues [46] are not found in any of the four Xog1p sequences (Fig. 2). If these Xog1p sequences can indeed bind LL-37, then LL-37 may indirectly reduce Xog1p activity by inducing a conformational change rather than by binding to a site(s) involved in Xog1p catalysis. Therefore, additional physical characterization of the LL-37/Xog1p interaction(s) is necessary and is underway in our laboratory.

Even when both *XOG1* alleles had been deleted and the cells were in the logarithmic-growth phase, neither the cellular exoglucanase activity nor cell adhesion was completely abolished (Fig. 5E, 5C, respectively). These results suggest that other cell-wall enzymes or polysaccharides compensate by participating in cell-wall remodeling [45], [62], which might explain why cell adhesion and exoglucanase activity were less affected in cells during logarithmic growth (Fig. 5E) than when cells were in the stationary phase (Fig. 5B). As deletion of *XOG1* did not completely abolish LL-37 binding (Fig. 5D), another protein(s) or polysaccharides probably also binds LL-37. Three CWPs that might bind LL-37 were found by far-western blotting (Fig. 1B). Two have molecular masses of 45∼50 kDa, and one has a mass of ∼60 kDa. However, we focused on only Xog1p, which has a molecular mass of ∼45 kDa, as it is primarily responsible for the polysaccharide composition of the cell wall. The identification of other possible LL-37-targeted protein(s) will be performed in the future.

In summary, we showed that LL-37 prevented *C. albicans* colonization by inhibiting the attachment of *C. albicans* to polystyrene and epithelial cell surfaces via interacting with carbohydrates and/or cell-wall proteins. Using phage display, we identified cell-wall Xog1p as a LL-37 binding target, which may play a role in adhesion inhibition mediated by LL-37. We have now shown that this inhibition may partially cause by LL-37 binding to Xog1p, followed by reducing Xog1p activity. Consequently, cell-wall remodeling might be interfered. We also showed that Xog1p itself is also involved in *C. albicans* adhesion through direct or indirect ways. Thus, we proposed that if a certain CWP is bound by LL-37, CWP is assumed to have potential to involve in cell adhesion. Given our observations, LL-37 may be a useful tool with which to screen for other CWPs involved in *C. albicans* adhesion. Because the cells of higher eukaryotes do not have a cell wall, Xog1p could perhaps be efficaciously targeted by monoclonal antibodies or short peptides to block fungal adhesion during infection.

## Materials and Methods

### Peptides and reagents

LL-37 (LLGDFFRKSKEKIGKEFKRIVQRIKDFLRNLVPRTES) and the biotinylated BA-LL37 were synthesized by MDBio, Inc. (Taipei, Taiwan). The results of HPLC and mass spectrometry showed that the peptides were 95% pure. All reagents were from Sigma-Aldrich (St. Louis, Mo) unless otherwise indicated.

### *C. albicans* strains, growth media, and growth conditions

Table 1 lists *C. albicans* strains used in this study. The cells were maintained at −80°C and plated onto YPD agar (1% yeast extract, 2% Bacto-Peptone, 2% glucose, and 1.5% agar) before each experiment. A single colony from a plate was inoculated in YPD broth and incubated at 30°C overnight (∼14 h). This culture was then sub-cultured in YPD broth for ∼2.5 h to reach logarithmic-growth phase. For LL-37 treatment, cells were washed twice with phosphate-buffered saline (PBS), collected by centrifugation, and suspended in Gibco RPMI-1640 medium (Invitrogen, Carlsbad, CA) or PBS.

**Table 1:** Strains of *Candida albicans* used in this study.

Strain	Parent	Genotype	Source
SC5314		Wild type	Gillum et al., 1984
PWTXA7	SC5314	*XOG1*/*xog1*Δ::*SAT1-FLIP*	This work
PWTXB73	PWTXA7	*XOG1*/*xog1*Δ::*FRT*	This work
PWTXC7311	PWTXB73	*xog1*Δ::*FRT*/*xog1*Δ::*SAT1-FLIP*	This work
PWTXD73115	PWTXC7311	*xog1*Δ::*FRT*/*xog1*Δ::*FRT*	This work
PWTXE3	PWTXD73115	*xog1*Δ::*XOG1*-*SAT1-FLIP*/*xog1*Δ::*FRT*	This work
PWTXF39	PWTXE3	*xog1*Δ::*XOG1*-*FRT*/*xog1*Δ::*FRT*	This work
PWTXG3918	PWTXF39	*xog1*Δ::*XOG1*-*FRT*/*xog1*Δ::*XOG1*-*SAT1-FLIP*	This work
PWTXH39184	PWTXG3918	*xog1*Δ::*XOG1*-*FRT*/*xog1*Δ::*XOG1*-*FRT*	This work
PWTEA7	PWTXH39184	*EXG2*/*exg2*Δ::*SAT1-FLIP*	This work
PWTEB71	PWTEA7	*EXG2*/*exg2*Δ::*FRT*	This work
PWTEC711	PWTEB71	*exg2*Δ::*FRT*/*exg2*Δ::*SAT1-FLIP*	This work
PWTED7113	PWTEC711	*exg2*Δ::*FRT*/*exg2*Δ::*FRT*	This work

### LL-37/*C. albicans*–binding assay

The binding of LL-37 to *C. albicans* was assayed as described [21], [63]. Briefly, the CWPs were removed by proteinase K (1 mg/ml) at 30°C for 1 h, then cells (6×10^6^) were mixed with 10-µg BA-LL37 in 750-µl PBS and incubated at 4°C overnight. The extent of binding was assessed by flow cytometry (FACSCalibur equipped with a diode laser, excitation at 488 nm; BD Bioscience, San Jose, CA) with SA-DTAF detection (3 µg/reaction; Jackson ImmunoResearch, West Grove, PA). The fluorescence emission from the cells was passed through an FL1 filter (515–545 nm), and the fluorescence intensity was recorded. The amount of LL-37 bound to *XOG1* and *EXG2* cells was normalized to mean fluorescence index of the wild type strain and reported as percentages.

### CWP extraction and western blotting

*C. albicans* cell-wall extracts were fractionated as described [64], [65], with modifications. Briefly, 3×10^9^ cells were incubated in PRMI-1640 at 37°C for 30 min. The cells were washed twice with PBS and then incubated in 50 mM Tris-HCl, pH 7.8, 1% β-mercaptoethanol (β-ME) at 37°C for 30 min. The supernatant was collected by centrifugation (1,000× *g*) for 10 min and designated the β-ME cell-wall fraction. The β-ME-treated cells were washed with 1 M sorbitol and suspended in 1 M sorbitol, 0.1 M sodium citrate, pH 5.8, 25 mM EDTA, and 2 U β-glucanase (49101; Sigma-Aldrich) at 30°C for 1 h. This solution was centrifuged at 1,000× *g* for 10 min, and the supernatant was designated the β-glucanase extract. Proteins from the two extracts were electrophoresed through SDS (MDBio, Inc.) 10% polyacrylamide gels (40% acrylamide/bis solution; MDBio, Inc.) and then transferred to polyvinylidene difluoride membranes (Pall Corporation, Port Washington, NY).

After transfer of the proteins, the membranes were blocked with 3% non-fat milk at room temperature for 2 h. For Xog1p detection, the membranes were probed with mouse monoclonal anti-His_6_ (Roche Applied Science, Indianapolis, IN) or rat polyclonal anti-Xog1p and visualized using goat anti-mouse IgG-HRP (Santa Cruz Biotechnology, Inc., Santa Cruz, CA) or goat anti-rat IgG-HRP (Jackson ImmunoResearch). To detect LL-37-binding proteins by far-western blotting, BA-LL37 (1.1 µM) in 0.1% PBST [PBS, 0.1% (v/v) Tween-20, without BSA or dried milk] served as the primary probe, and HRP–conjugated streptavidin (SA-HRP; Zymed Laboratories, San Francisco, CA) was used for visualization with ECL kit reagents (PerkinElmer Life Sciences, Wellesley, MA) according to the manufacturer's instructions.

### Identification of LL-37-binding proteins by phage-display biopanning

Biopanning was performed with a library generated by Ph.D.-12™ Phage Display Peptide Library reagents (New England BioLabs, Ipswich, MA) according to the manufacturer's instructions. LL-37 (15 µg in 150 µl 0.1 M NaHCO_3_, pH 8.6) was coated onto wells of 96-well microtiter plates (Nunc™, Rochester, NY) and incubated overnight at 4°C with gentle agitation. Each well was filled with blocking buffer (0.1 M NaHCO_3_, pH 8.6, 5 mg/ml BSA, 0.02% NaN_3_) for 1 h at 4°C and then washed with 0.1% TBST [TBS, 0.1% (v/v) Tween-20]. For the first round of panning, 10 µl of the phage suspensions (1.5×10^11^ virions in 100 µl of 0.1% TBST) was added to the LL-37-coated wells and incubated for 1 h at room temperature with gentle agitation. Unbound phages were discarded, and the wells were washed 10 times with 0.1% TBST. LL-37 samples (10 µg in 100 µl of TBS) were added to the wells and incubated for 1 h at room temperature to elute the bound phage. For the second and third rounds of biopanning, the aforementioned procedures were repeated, except that the eluates from the first and second rounds were used as input for the second and third rounds, respectively. In addition, the concentration of Tween-20 in the TBST-wash buffer was increased to 0.5% (v/v).

After each round of biopanning, eluted phages were amplified in and titered using *E. coli* ER2738. Samples of *E. coli* in Luria-Bertani (LB, 20 ml) broth were infected with the eluted phages and incubated for 4.5 h at 37°C. Cultures were centrifuged two times (10,000× *g*) for 10 min at 4°C by transferring the supernatant to a new tube after first centrifugation. To precipitate the phages, the upper 80% of the supernatants was transferred to new tubes and incubated with 1/6 volume of 20% (w/v) polyethylene glycol-8000, 2.5 M NaCl at 4°C overnight. The solutions were centrifuged at 10,000× *g* for 15 min at 4°C, and the pelleted phages were suspended in 1 ml TBS. The samples were centrifuged at 10,000× *g* for 5 min at 4°C, and the supernatants were precipitated as described above for 1 h at 4°C to isolate the phages. Finally, the phages were harvested by centrifugation at 10,000× *g* for 10 min at 4°C and suspended in 200 µl of TBS. After centrifugation at 10,000× *g* for 1 min at 4°C, the supernatants were collected and used as the amplified phage samples.

Eluates of biopanned and amplified phages that had been serially diluted 10-fold starting with 10-µl volumes were each added into 200 µl of mid-logarithmic–growth phase *E. coli* ER2738 cultures. The infected *E. coli* cultures were each suspended in top agarose (LB broth, 0.7% agarose) and poured onto LB agar plates that contained 50 µg/ml isopropyl β-d-thiogalactoside (MDBio, Inc.), 40 µg/ml 5-bromo-4-chloro-3-indolyl-β-d-galactoside (MDBio, Inc.). The plates were incubated overnight at 37°C. Phage titers were calculated by counting the number of plaques and multiplying by the serial-dilution factor.

### Characterization of cloned, LL-37-binding phage

After three rounds of biopanning, sequencing templates were rapidly purified, and the sequences of selected LL-37-binding peptides were determined according to the manufacturer's instructions (New England BioLabs) and [66]. Twenty isolated blue plaques were transferred to *E. coli* ER2738 cultures (1 ml each). The cultures were incubated at 37°C for 4.5 h and centrifuged at 10,000× *g* for 30 sec. The supernatants were centrifuged again under the same conditions, and 500-µl of each supernatant was added to 200-µl 20% (w/v) polyethylene glycol-8000, 2.5 M NaCl. After incubation at room temperature for 10 min, the supernatants were centrifuged twice for 10 min, and the pellets were suspended in 100 µl of 10 mM Tris-HCl, pH 8.0, 1 mM EDTA, 4 M NaI, after which 250 µl absolute ethanol was added. Phage DNA was collected by a 10-min centrifugation at 10,000× *g* at room temperature, washed with 70% ethanol, and dissolved in 30 µl H_2_O. The isolated DNA was sequenced using the −96 gIII sequencing primer (5′-CCCTCATAGTTAGCGTAACG-3′).

Additionally, 2.5 µl of each amplified clone that had been sequenced was added into a 5-ml *E. coli* ER2738 culture to be amplified again as described above. ELISA plates were coated with LL-37 and blocked with blocking buffer before adding phage. Phages were prepared as four-fold serial dilutions in 200 µl 0.1% TBST per well, with 2×10^10^ virions in the first well and 1.2×10^6^ virions in the last well. After incubation and washing as described for biopanning, mouse monoclonal anti-M13 (Santa Cruz Biotechnology, Inc.) was diluted 1∶2,000 in 200 µl blocking buffer and added into each well. The plates were incubated at room temperature for 1 h and then washed six times with 0.1% TBST. HRP-conjugated anti-mouse IgG, diluted as described above, was added into the wells, which were then incubated for 1 h at room temperature. Each well was then washed six times with 0.1% TBST. Binding was detected using 3,3′,5,5′-tetramethylbenzidine with the absorbance at 450 nm.

### Searching for the potential LL-37 interacting proteins from Candida Genome Database (CGD)

The consensus pattern identified from phage-display biopannings, ΦHWXΦΦXΦXΦ, was used as a reference segment, and transformed to “JHWXJJXJXJ”, where J means any hydrophobic residue, and X represents any amino acid residue. The transformed pattern was input into PatMatch of the CGD. In order to increase the matching hits, any of the continuous 4 residues matched with the sequences in CGD were chosen. Then, the chosen sequences representing proteins located at cell wall were particularly selected. As results, the phage clones has 3 perfect matches to the peptide segments of Xog1p, including Xog1p_90–93_, Xog1p_134–140_, and Xog1p_398–403_. In the comparison of the whole Xog1p sequence with the consensus pattern, another segment, Xog1p_108–112_, was also identified as a potential region for LL-37 binding.

### ELISA for LL-37/Xog1_90–115_ association

The extent of binding of Xog1_90–115_ (residues 90–115 from Xog1p, YHWTQTLGKEAASRILQKHWSTWITE), which was synthesized by MDBio, Inc., to LL-37 was determined by ELISA. Xog1_90–115_ (5 µg) was coated onto the wells of 96-well microtiter plates. After wells were blocked and washed as for the biopanning procedure, BA-LL37 (0.01–5 µg/ml in 100 µl of 0.1% TBST) was added to individual wells. After incubation and washing, the BA-LL37 that had bound Xog1_90–115_ was detected using SA-HRP and 3,3′,5,5′-tetramethylbenzidine. The absorbance at 450 nm was measured using a VICTOR3 Multilabel plate reader (PerkinElmer, Inc.).

### Expression, purification, and refolding of recombinant Xog1p

*XOG1* was amplified via PCR from *C. albicans* SC5314 genomic DNA using the primers 5′-ATATCATATGGGACATAATGTTGCTTGG-3′ and 5′-ATATCTCGAGGTGAAAGCCACATTGGTTTG-3′ (the *Nde*I and *Xho*I sites are underlined and doubly underlined, respectively). The DNA fragment carrying *XOG1* was isolated by digestion with *Nde*I *and Xho*I, ligated into pGEM-T Easy, sequenced, and cloned into pET23a(+) (pET23-*XOG1*).

For the expression of rXog1p, pET23-*XOG1* was transformed into *E. coli* BL21(DE3)pLysS. A colony was added into 15 ml LB broth containing 100 µg/ml carbenicillin and 50 µg/ml chloramphenicol, and the culture was incubated at 37°C and 200 rpm overnight. This culture was added into 500 ml of LB broth that also contained carbenicillin and chloramphenicol at 37°C and incubated until its OD_600_ reached 0.5–0.8. rXog1p expression was induced with isopropyl β-d-thiogalactoside (0.5 mM, final concentration), and the culture was incubated for an additional 5 h. Cell pellets were harvested by centrifugation, suspended in 15-ml PBS, and sonicated. The insoluble fraction was isolated by centrifugation at 10,000× *g* at 4°C for 10 min.

The inclusion bodies contained in the insoluble fraction were dissolved in 10 ml binding buffer (6 M urea, 0.5 M NaCl, 20 mM Tris-HCl, pH 7.5) and incubated overnight at 4°C. After centrifugation at 10,000× *g* for 30 min at 4°C, the supernatant was processed using Ni^2+^-chelating chromatography (Promega, Madison, WI). Unbound proteins were removed sequentially with binding buffer and then with 10 mM imidazole in binding buffer. rXog1p was eluted with a linear gradient of 50–300 mM imidazole in binding buffer. The purity of rXog1p was assessed after electrophoresis through an SDS 12% polyacrylamide gel and staining with Coomassie Blue (data not shown).

To refold rXog1p, reduced glutathione (80 mM, final concentration) was added into a solution of purified rXog1p, which was then incubated at room temperature for 30 min. Denatured rXog1p was refolded by rapid dilution (100 fold) into 0.1 M Tris-HCl, pH 7.5, containing 10% glycerol, 1 mM EDTA, 0.5 M l-arginine, protease inhibitors (1 mM phenylmethylsulfonyl fluoride, 40 µM benzamidine, 40 µg/ml aprotinin, 20 µg/ml leupeptin, 20 µM 4-(2-aminoethyl) benzenesulfonyl fluoride hydrochloride), and oxidized glutathione so that the final reduced/oxidized glutathione ratio was 4∶1. The protein was incubated at 4°C with very slow stirring for 24 h, and then was concentrated using a Centricon system (10K MWCO, Millipore, Billerica, MA) at 4°C. The final protein concentration was determined using BCA assay reagents (Thermo Scientific).

### Preparation and purification of polyclonal antibodies against *C. albicans* Xog1p

All animal studies were approved by the Institutional Animal Care and Use Committees of Animal Technology Institute Taiwan (approval number 10003). Two female Sprague-Dawley rats (BioLasco Taiwan Co., Ltd.) were injected subcutaneously with 200 µl of an emulsion that contained 100 µg rXog1p in PBS and an equal volume of Freund's complete adjuvant (primary immunization) or an equal volume of Freund's incomplete adjuvant (booster immunization). Beginning 2 weeks after primary immunization, four boosters were given at 3-week intervals over 3 months. Serum from each immunized rat was isolated by centrifugation at 4,000× *g* for 10 min. Pre-immune serum was collected from each un-immunized rat, pooled, and processed in the same manner.

Anti-Xog1p and mock antisera were further purified using Montage® Ab Purification kit reagents (Millipore). The Xog1p antiserum was filtered through a 0.2-µm syringe filter to remove debris and then mixed 1∶1 (v/v) with binding buffer A (1.5 M glycine/NaOH buffer, 3 M NaCl, pH 9.0). A spin column was fabricated using a centrifuge tube that was filled with protein A medium (PROSE-A, Millipore); the column was equilibrated with 10 ml binding buffer A by centrifugation at 500× *g* for 5 min. The filtered serum (10 ml) was then added into the spin column and centrifuged at 150× *g* for 20 min at 4°C. After removing the supernatant, another 10 ml of filtered serum was loaded and centrifuged at 150× *g* for 20 min at 4°C. Then, the spin column was washed with binding buffer A to remove unbound contaminants and centrifuged at 500× *g* for 2 min at 4°C. Anti-Xog1p was eluted with 0.2 M glycine/HCl, pH 2.5, and the eluate was immediately neutralized with 1 M Tris-HCl, pH 9.0. Eluted anti-Xog1p was concentrated using an Amicon® Ultra-30K system (Millipore). Finally, NaN_3_ [0.1% (w/v), final percentage] and glycerol [50% (v/v), final percentage] were added, and the concentrated antiserum was stored at −20°C.

### Interaction of LL-37 and rXog1p

Streptavidin-agarose (10 µl), BA-LL37, and rXog1p in 500 µl TBS were gently shaken at room temperature for 2 h and then pelleted. The pellets were washed six times with 1 ml of TBS. After the last wash, the pellets were suspended in sample buffer that contained SDS and β-ME and heated at 100°C for 10 min. The LL-37/rXog1p complexes were identified after electrophoresis through Tricine SDS 15% polyacrylamide gels and staining with Coomassie Blue.

### *C. albicans* adhesion to polystyrene

*C. albicans* adhesion to uncoated, flat-bottom, polystyrene wells of 24-well plates (Orange Scientific, Braine-I'Alleud, Belgium) was as described [21], [67], [68]. The cells were harvested, washed with PBS, and suspended in RPMI-1640 medium at a density of ∼6×10^7^ cells/ml. For competition assays, LL-37 was mixed with Xog1_90–115_ or rXog1p and the cells. To assess the effect of gene deletion on cell adhesion, the *C. albicans* strains listed in Table 1 were used. To assess the effect of Xog1p antiserum on cell adhesion, different concentrations of anti-Xog1p serum were added to cells. Rabbit polyclonal antibody that recognizes numerous proteins in a soluble *C. albicans* extract (Biodesign International, Saco, ME) was also used. Next, 250 µl of the cell suspensions were each transferred into a well of a 24-well flat-bottom plate and incubated at 37°C for 30 min at 100 rpm. The metabolic activity of the sessile cells was then measured by detecting the reductive adduct of XTT [69]. Briefly, cells were washed three times with PBS to remove floating cells. The remaining cells were incubated with 300 µl XTT (1 mg/ml) and 0.6 µl menadione (1 µM) in PBS per well at 37°C for 20 min. The absorbance at 490 nm for each sample was measured using a VICTOR3 Multilabel plate reader. The relative percentage of cells was calculated as: mean absorbance of [each treatment (*XOG1*, *EXG2* mutant strains or anti-Xog1p, anti-*C. albicans* serum)]/[no treatment (wild type or pre-immune serum)]×100%. All assays were performed in duplicate or triplicate and repeated two to three times.

### Glucanase activity assay

Cells for the β-glucanase activity assay were prepared as described [45]. Briefly, 1×10^9^ cells were centrifuged and the supernatant was removed. The pellet was suspended in 500 µl of 50 mM sodium acetate, pH 5.5. In addition, a β-ME-treated cell-wall extract was prepared as described above but was then suspended in 500 µl of 50 mM Tris-HCl, pH 7.8, 1% β-ME. A 200-µl sample of cells or a 200-µl sample of the cell-free extract were separately transferred to two Eppendorf tubes. A solution of PNPG (5 mg/ml) in 50 mM sodium acetate, pH 5.5 (200 µl) was added into one of the tubes that contained cells or one that contained cell-free extract. Duplicate samples to which PNPG was not added served as background control of each sample. In addition, the buffer same as that in the test samples was also included as a blank in this assay, including one tube with PNPG and the other without PNPG. All samples were incubated at 37°C for 3 h, and then 1 ml of 1 M Na_2_CO_3_ was added to each tube to stop the reactions. Hydrolysis of PNPG was measured spectrophotometrically at 410 nm, and the activity of each sample after PNPG hydrolysis, absorbance of PNPG-free sample was subtracted from absorbance of PNPG-containing sample. The relative glucanase activity was calculated as: [(absorbance for each LL-37-treated or mutant strain)/(absorbance for the untreated or wild type sample)]×100%. The assays were performed three to four times for each strain and treatment.

### Cell susceptibility to agents that interrupt cell-wall integrity

Cells were grown as described above and suspended in RPMI-1640 at 1.2×10^7^ cells/ml. After incubation with different concentrations of LL-37, 10-µl cell samples that had undergone 10-fold serial dilutions were spotted onto YPD agar plates that contained 10 µg/ml calcofluor white or 15 µg/ml congo red. Cell viability was recorded after incubation at 30°C for 20 h. This assay was performed independently at least three times.

### Construction of *C. albicans* mutant strains and Southern blotting

To generate the *C. albicans XOG1*- and *EXG2*-null strains, the *SAT1*-flipper method was used [48]. An *XOG1*-deletion cassette was constructed as follows: an *Apa*I-*Xho*I fragment containing a *C. albicans XOG1* upstream sequence (positions −726 to −334) was amplified using SC5314 genomic DNA and the primers XOG1-1 [5′-ATATGGGCCCCAAACACAATCGCAAATTGA-3′] and XOG1-2 [5′- ATATCTCGAGATTGCAAGCGACTTGGTCTT-3′] (the *Apa*I and the *Xho*I sites are singly and doubly underlined, respectively). A fragment that contained an *XOG1* downstream region from positions +1761 to +2241 was amplified with the primers XOG1-3 [5′-ATATCCGCGGTGCTTTGTTCTTGATTGCTG-3′] and XOG1-4 [5′-ATATGAGCTCCACATTGCCTGAAGTCGTTG-3′] (the *Sac*II and *Sac*I sites are underlined and double underlined, respectively). The *XOG1* upstream and downstream fragments were cloned into pSFS2A (a kind gift from Professor J. Morschhauser, University of Wurzburg, Germany) to generate pXOG1M2. For *EXG2* deletion, a DNA fragment containing a region upstream of *C. albicans EXG2* was amplified with primers EXG2-1 [5′-ATATGGGCCCGAAGCCGAATCCAAACAAAA-3′] and EXG2-2 [5′-ATATCTCGAGTGACAGTTGGTGCTCCCTTA-3′] (the *Apa*I and the *Xho*I sites are underlined and doubly underlined, respectively). A fragment containing the *C. albicans EXG2* downstream region was amplified with primers EXG2-3 [5′-ATATCCGCGGATCCGGTGTTGTTGGTTCAT-3′] and EXG2-4 [5′-ATATGAGCTCCCTTTTTGTTTGGGGTAGCA-3′] (the *Sac*II and *Sac*I sites are underlined and double underlined, respectively). Both DNA fragments were cloned into pSFS2A to generate pEXG2M2. The DNA fragments carrying the regions that flanked *XOG1* or *EXG2* and the *SAT1* flipper cassette were independently isolated from pXOG1M2 or pEXG2M2, respectively, by digestion with *Apa*I and *Sac*I. Following transformation into *C. albicans* SC5314, each cassette was integrated into the chromosome by homologous recombination of the *XOG1* or *EXG2* flanking sequences. Transformants were selected for nourseothricin resistance and subsequently grown for 2 days in YPM (1% yeast extract, 2% Bacto-peptone, 2% maltose) medium to induce recombinase, which excised the *SATI* marker. The remaining intact *XOG1* and *EXG2* alleles in the *XOG1*/*xog1*Δ or *EXG2/exg2*Δ strains were then each inactivated. Two independently generated heterozygous and homozygous *XOG1*- and *EXG2*-deletion strains were used initially in the cell adhesion assays, and then only one clone of each was used for further studies.

For re-integration of *XOG1* into the original loci of the *xog1*Δ/*xog1*Δ strain, the *Apa*I- *Xho*I fragment that contained the complete *XOG1* gene and the 0.33-kb upstream flanking sequence of *XOG1* was amplified with the primers XOG1-1 and XOG1-5 [5′-ATATCTCGAGTCAGTGAAAGCCACATTGGT-3′] (the *Xho*I sites is double underlined) and substituted for the *XOG1* upstream flanking sequence in pXOG1M2 to generate plasmid pXOG1M3. Sequential reintegration of *XOG1* was also performed by the *SAT1*-flipper method. Strain construction was verified by PCR and Southern and western blotting using standard methods [70].

### RNA isolation and reverse transcriptase-PCR

*C. albicans* cells were grown in YPD overnight. Then total RNA was isolated using TRI reagent® (Ambion, Inc.), and the RNA was treated with TURBO™ DNase (Ambion, Inc.) according to the manufacturer's instructions. Total RNA (1 µg) was reverse transcribed into single-stranded cDNA with M-MLV reverse transcriptase (Promega) and oligo(dT)_18_ (MDBio, Inc.). *XOG1* or *EXG2* cDNA and *ACT1* or *EFB1* cDNA (as internal controls) were PCR amplified with the primers 5′-CAGTTGACGAATATCACTGGACA-3′ (forward) and 5′-AATATCCAACAATGGTTGACAGG-3′ (reverse) for *XOG1*, and 5′-CAGTTACGGTCTGTGTCCAGTGTAG-3′ (forward) and 5′- GGACACACATGGAGGTTTAAAGAAG-3′ (reverse) for *EXG2*. The primers used for *ACT1* were 5′-GGCTGGTAGAGACTTGACCAACCATTTG-3′ (forward) and 5′-GGAGTTGAAAGTGGTTTGGTCAATAC-3′ (reverse), and for *EFB1* were 5′-ATTGAACGAATTCTTGGCTGAC-3′ (forward) and 5′-CATCTTCTTCAACAGCAGCTTG-3′ (reverse). Each primer was 0.5 µM. Each reaction mixture was first denatured at 95°C for 10 min. The PCR program consisted of 30 cycles of 95°C for 30 sec, 55°C for 30 sec, and 72°C for 30 sec, followed by a final 10-min incubation at 72°C. PCR products were visualized by SYBR® Safe (Invitrogen) staining after agarose gel electrophoresis.

### Statistical analysis

Data were assessed for statistical significance by the two-tailed Student's *t*-test.

### Accession numbers

Information concerning the genes/proteins used in this study can be obtained at the Candida Genome Database (http://www.candidagenome.org). The genes and their corresponding open reading frame numbers (in parentheses) are: *XOG1* (orf19.2990) and *EXG2* (orf19.2952).
